# Effects of Ozone Gas and Slightly Acidic Electrolyzed Water on the Quality of Salmon (*Salmo salar*) Fillets from the Perspective of Muscle Protein

**DOI:** 10.3390/foods13233833

**Published:** 2024-11-28

**Authors:** Yun-Fang Qian, Lu Sun, Jing-Jing Zhang, Cheng-Jian Shi, Sheng-Ping Yang

**Affiliations:** 1 College of Food Science & Technology, Shanghai Ocean University, Shanghai 201306, China; yfqian@shou.edu.cn (Y.-F.Q.); 18763069701@163.com (L.S.); jjingzhang2022@163.com (J.-J.Z.); 19962263324@163.com (C.-J.S.); 2 Shanghai Engineering Research Center of Aquatic Product Processing & Preservation, Shanghai 201306, China

**Keywords:** cathepsins, calpain, protein oxidation, protein degradation, Fourier transform infrared spectroscopy

## Abstract

To elucidate the mechanisms of ozone gas (OG) and slight acid electrolyzed water (SA) on the quality changes in texture, water-holding capacity, and softening of salmon, the bacterial growth, total volatile basic nitrogen, thiobarbituric acid reactive substance, a* value, texture properties, carbonyl content and free sulfhydryl content, myofibrillar fragmentation index, and proteolytic activities of salmon treated by OG (1 mg/m^3^ for 10 min) and SA (ACC 30 mg/L, 5 min) individually and in combination were studied. The results showed that total viable counts of SA + OG (dipped in SAEW for 5 min, followed by exposure to ozone for 10 min) was about 3.36 log CFU/g lower than the control (CK) (dipped in distilled water for 5 min) on day 10. Further studies indicate that at the end of storage, the hardness of SA + OG fillets only decreased by 33.95%, while the drip loss and myofibrillar fragmentation index (MFI) were the lowest (i.e., 14.76% and 101.07). The activity of cathepsin D was extensively inhibited by SA + OG, which was only 2.063 U/g meat at the end. In addition, the carbonyl content was 1.90 μmol/g protein, and the free sulfhydryl content was 39.70 mg/mL in the SA + OG group, indicating that protein oxidation was also effectively inhibited. Correlation analysis shows that bacteria and endogenous proteases are the main causes of protein degradation. Overall, the combination of OG and SAEW is an effective way to maintain the muscle quality of salmon by inhibiting bacterial growth and endogenous enzymes.

## 1. Introduction

Salmon (*Salmo salar*) is an important commercial seafood that is commonly consumed raw in dishes such as sushi and sashimi globally [1]. According to the Salmon Market Report—Size and Price Forecast by Mordor Intelligence (https://www.mordorintelligence.com/industry-reports/salmon-market, accessed on 22 November 2024), the salmon market size is projected to be USD 33.5 billion in 2024 and is expected to reach USD 49.39 billion by 2029, with a compound annual growth rate (CAGR) of 8.07% during the forecast period (2024–2029). Therefore, there are high requirements for its safety and taste quality [2]. The main production area of salmon is in the Atlantic Ocean, and it usually needs to be transported by air freight through cold chain logistics, which covers limited shelf life (about 6~11 days) to a shorter sales period [3]. The main reasons for the deterioration of salmon quality include microbial spoilage, protein degradation, and the oxidation of lipids and proteins [4,5,6]. Therefore, effective preservation methods are crucial.

In recent years, ozone technology has attracted attention in the preservation of seafood products, including salmon [7]. Ozone (O_3_) is a powerful oxidant. It is penetrating, short half-life, low-residue, and ecological-friendly, which cannot only kill bacteria in food but also decontaminate viruses, including coronavirus and norovirus [8,9,10]. In addition, ozone treatment has a certain effect on removing fishy smells, making the smell of raw salmon fillet more acceptable and extends the shelf life of refrigerated salmon by about 4 days [7]. Moreover, with appropriate treatment methods, ozone does not promote lipid oxidation but rather has an inhibitory effect to some extent [11].

Another preservation method that has gained attention in recent years is slight acidic electrolyzed water (SAEW). It acts as a bactericide by generating HClO and lowering pH [12]. There is a possible application scenario for AEW by an alteration of tap water for cleaning during the harvesting, processing, and the washing of fruits, vegetables, seafood, and meat products is very feasible [13,14,15,16,17].

Currently reported researches mainly focus on the bactericidal effects of ozone and SAEW [18,19]. However, the mechanisms of OG and SAEW on the quality changes in texture, water-holding capacity, and the softening of salmon is still unclear. Therefore, this article first aims to compare the changes in the microbiological and physicochemical properties of salmon treated with SAEW and ozone alone or in combination during storage. It particularly focuses on the effects of salmon protein degradation, oxidation, and texture. By analyzing the correlation between endogenous enzymatic activity, myofibrillar fragmentation index, and texture, we preliminarily explored the mechanisms of ozone and SAEW in maintaining the texture quality of salmon. This study provides reference for research on sanitation and rinsing treatment methods for seafood.

## 2. Materials and Methods

### 2.1. Preparation of Salmon Fillets

Chilled adult salmon fillets (*Salmo salar*, 96 pieces) were purchased from local supermarket (Pudong District, Shanghai, China). Each piece of salmon was about 10 × 8 × 2 cm in volume and weighed about 200 g. The salmon were transported to the laboratory within 30 min in a clean container with ice flakes (0 °C).

Slightly acidic electrolyzed water (SAEW, pH 5.86; oxidation–reduction potential (ORP), 911 mV; ACC, 30 mg/L) was produced by an electrolysis device (FX-SWS100 Micro Acid Water Electrolysis Generator, Antai, Yantai, China). The fish were dipped in the SAEW for 5 min [12]. Ozone gas (OG, 1 mg/m^3^) was produced through an ozone generator (FH-CYJ1510A-W Ozone Generator, Shanghai Fenghua Optoelectronics Technology Co., Ltd., Shanghai, China), which is based on the corona discharge method through which O_2_ in the air is used to generate O_3_. According to previous experimental studies [7], the ozone concentration was selected as 1 mg/m^3^ and the treatment lasted for 10 min.

The salmon were randomly allocated to four groups: (1) dipped in distilled water for 5 min (CK); (2) dipped in SAEW for 5 min (SAEW); (3) dipped in distilled water for 5 min, followed by exposure to OG for 10 min (OG); (4) dipped in SAEW for 5 min, followed by exposure to ozone for 10 min (SA + OG). The salmon pieces were then placed in polyethylene bags (thickness: 0.1 mm) and stored at 4 °C until further analysis at 2-day intervals over 10 days.

### 2.2. A Global Study on the Effect of OG and SAEW on Salmon

#### 2.2.1. Microbial Analysis

Twenty-five grams of salmon flesh was dispersed evenly in 225 mL sterilized saline. Total viable counts (TVCs) and psychrotrophic bacterial counts (PBCs) were determined using PCA agar (P9270, Beijing Solarbio Science & Technology Co., Ltd., Beijing, China) after cultivation at 30 °C for 72 h and 4 °C for 10 days, according to the method of Yu, et al. [20].

#### 2.2.2. Total Volatile Basis Nitrogen (TVB-N) and Thiobarbituric Acid Reactive Substance (TBARS)

Total volatile basis nitrogen (TVB-N) was determined using a FOSS Kjeltec 8400 analyzer unit (Foss Analytical Co., Ltd., Höganäs, Sweden) and thiobarbituric acid reactive substance (TBARS) was determined by the method of Qian, Zhang, Liu, Ertbjerg, and Yang [7].

#### 2.2.3. Redness

The redness value (a∗) of salmon was carried out using a benchtop colorimeter (YS6060, Shenzhen Threenh Technology Co., Ltd., Shenzhen, China) referred to in Ye, et al. [21]. Each batch was repeated in triplicate.

#### 2.2.4. TCA-Soluble Peptides

The TCA-soluble peptide concentration in salmon was determined by taking 2.0 g of minced flesh with 18 mL of 5% (*w*/*v*) trichloroacetic acid (TCA, W12564, Shanghai Yuanye Biotechnology Co., Ltd., Shanghai, China) solution. The following process was conducted according to the method of Sriket, et al. [22].

### 2.3. Muscle Properties Analysis

#### 2.3.1. Texture

The sample was cut to small blocks (10 mm × 10 mm × 10 mm). The texture was measured according to the method of Wang, et al. [23]. The speed before measurement was 3 mm/s; measurement speed was 1 mm/s; post measurement rate was 1.00 mm/s; sample deformation was 50%; and trigger point load was 5 g. Each sample was required to be repeated three times.

#### 2.3.2. Drip Loss

The determination of drip loss was conducted according to the method of Ye, Ding, Zhu, Wu, Hu, and Li [21]. The mean values were calculated from three replicates.

### 2.4. Microstructure of Muscle

The sample was cut (approximately 3 mm × 3 mm × 3 mmin size) from the back of the fillet and immersed in 3% glutaraldehyde, referring to the experimental method of Zhuang, et al. [24].

### 2.5. Determination of Protein Oxidation

#### 2.5.1. Carbonyl Content

The carbonyl content of myofibrillar protein was determined by a protein carbonyl content detection kit (BC1270-50T/24S, Beijing Solarbio Science & Technology Co., Ltd., Beijing, China). According to the kit instructions, the reagents provided in the kit were mixed with the sample in the tube. After completing all the reactions, the solution was transferred from the test tube to a quartz cuvette (UV-2100 UV-Vis Spectrophotometer, Unico Instrument Co., Ltd., Shanghai, China) and the absorbance was recorded at 370 nm.

#### 2.5.2. Free Sulfhydryl Content

According to the method of Bao, et al. [25], 1.0 g of meat was homogenized in 25 mL of 5% (*w*/*v*) SDS (T90576, Shanghai Yuanye Biotechnology Co., Ltd., Shanghai, China) and 0.1 M Tris-HCl (pH 8.0, T90537, Shanghai Yuanye Biotechnology Co., Ltd., Shanghai, China) for 30 s using a homogenizer (A25, Fokker Power Transmission (Shanghai) Co., Ltd., Shanghai, China). The homogenate was heated in a water bath at 80 °C for 30 min, and then cooled and filtered through filter paper (mesh, 30~50 microns). The absorbance A_280_ determined was used as the protein concentration.

The filtrate (0.5 mL) was mixed with 2 mL of Tris-HCl (pH 8.0, 0.1 M), and 0.5 mL of 10 mM DTNB (S30109, Shanghai Yuanye Biotechnology Co., Ltd., Shanghai, China). After incubation for 30 min at room temperature in the dark, the absorbance at 412 nm was measured. The results were calculated using the molar extinction coefficient (14,150 M^−1^·cm^−1^) of DTNB (nmol/mg protein).

#### 2.5.3. Fourier Transform Infrared Spectroscopy (FT-IR) Analysis

The extracted myofibril solution was freeze-dried for 72 h (Alpha 1–4 LSC plus, Christ, Osterode, Germany). After freeze-drying, the myofibril powder was pressed into thin flakes and then the secondary structure was measured using an FTIR spectrometer (Nicolet iS 10, Thermo Fisher Scientific Inc., Waltham, MA, USA). The parameters used for the measurements were as follows: 32 scans, 500 to 4000 cm^−1^ scan bands, and 4 cm^−1^ resolution. The data were analyzed with PeakFit software (Version 4.12).

### 2.6. Determination of Protein Degradation

#### 2.6.1. SDS-PAGE

SDS-PAGE profile of salmon protein was carried out according to Yu, et al. [26]. The sample of 10 μg of protein was taken for SDS-PAGE analysis using the electrophoresis apparatus (165-8001 Mini-PROTEAN, Bio-Rad Laboratories Inc., Hercules, CA, USA). Finally, staining and decolorization were performed.

#### 2.6.2. Myofibrillar Fragmentation Index (MFI)

The MFI of salmon during storage was measured according to Yang, et al. [27] with slight modifications. Specifically, 0.5 g of sample was homogenized with 30 mL of buffer (0.1 M KCl (B24593, Shanghai Yuanye Biotechnology Co., Ltd., Shanghai, China), 7 mM NaH_2_PO_4_ (S24161_,_ Shanghai Yuanye Biotechnology Co., Ltd., Shanghai, China), 18 mM Na_2_HPO_4_ (V33040_,_ Shanghai Yuanye Biotechnology Co., Ltd., Shanghai, China), 1 mM EDTA (S30020 EDTA, Shanghai Yuanye Biotechnology Co., Ltd., Shanghai, China), pH 7.0).

### 2.7. Proteolytic Activity

#### 2.7.1. Total Proteolytic Activity

The fish flesh was minced and homogenized with 3 volumes of buffer (pH 7.6), and then supernatant was obtained as the crude enzyme solution after centrifugation for 15 min at 4 °C at 5000× *g*, referring to Sriket, et al. [28] with slight modifications. The crude enzyme solution (200 μL) was homogenized with distilled water (200 μL), 625 μL of reaction buffer (0.2 mol/L Na_2_HPO_4_/0.1 mol/L citric acid (V32720, Shanghai Yuanye Biotechnology Co., Ltd., Shanghai, China), pH 5.0), and 200 μL of 10 g/L hemoglobin solution (R24183, Shanghai Yuanye Biotechnology Co., Ltd., Shanghai, China) at a time. The mixed solution was reacted in a constant temperature water bath at 50 °C for 15 min. After that, 200 μL of 50% TCA solution was added to stop the reaction and centrifuged at 5000× *g* for 10 min at 4 °C. The content of peptides in the supernatant was determined by the Lowry method. The entire experiment was conducted in triplicate.

#### 2.7.2. Endogenous Proteolytic Activity

Cathepsin B and cathepsin L activities were determined with cathepsin B (BBI D721170-0048, BBI life science Co., Shanghai, China) and cathepsin L activity kits (BBI D711388-0048, BBI life science Co., Shanghai, China). The salmon tissue (0.1 g) was weighed and mixed with 1 mL of PBS (pH 7.4). After homogenization under rotating speed 3000 r/min for 30 s, the mixture was centrifuged at 2500× *g* for 20 min at 4 °C. Finally, the supernatant was collected and analyzed following the protocol.

The cathepsin D activity was determined according to the method of Hagen et al. [29] with slight modifications. The hemoglobin (2.5% 100 μL) was mixed with 0.6 mL of 0.2 M citric acid buffer (pH 2.8), and 25 µL of crude enzyme solution. After incubation for 1 h at 37 °C, the reaction was stopped by adding 50 µL of 15% TCA solution. The mixture was centrifuged at 20,000× *g* for 5 min at room temperature, and the supernatant was collected. The stop solution of the blank control group was added before the crude enzyme solution. The content of peptides in the supernatant was determined by the Lowry method.

The determination method of calpain activity was referred to in the experimental method of Zhou, et al. [30]. The activity was determined using the Calpain Assay Kit (QIA120, Merck KGaA, Darmstadt, Germany).

### 2.8. Correlation Analysis

The correlation coefficients between the quality indicators were analyzed by calculating the Pearson value via Origin 2021. The *p* value was set as 0.05.

### 2.9. Statistical Analysis

Independent repetition of each experimental sample was conducted three times. The results were expressed as mean ± standard deviation. Significant difference was analyzed using SPSS 19.0 software (SPSS Inc., Chicago, IL, USA) via one-way analysis of variance and Duncan’s multiple range test (*p* < 0.05). Graphs and figures were created using Origin 2021 (OriginLab Inc., Hampton, MA, USA).

## 3. Results and Discussion

### 3.1. Effect of OG and SAEW on the Microbial and Physiochemical Properties of Salmon

**Figure 1:**
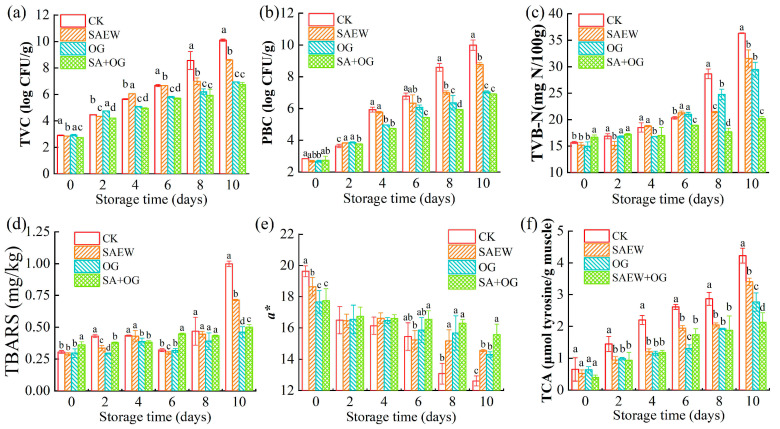
Changes in TVC (**a**), PBC (**b**), TVB-N (**c**), TBARS (**d**), a* value (**e**), and TCA-soluble peptides (**f**) of salmon fillets after treatment with ozone gas and slightly acidic electrolyzed water. (Different letters represent significant differences between four groups on the same day (*p* < 0.05)).

The overall effect of OG and SAEW on the microbial and physiochemical properties of salmon fillets was studied. The results of the TVC and PBC of salmon fillets during storage are described in Figure 1a,b. The TVC in the CK group increased from 2.91 log CFU/g to 6.67 log CFU/g during the first 6 days of storage, which was close to the microbial threshold (7.0 log CFU/g) [31]. On the other hand, the TVC of OG and SA + OG were only about 6.95 log CFU/g and 6.74 log CFU/g on day 10. The growth tendency of PBC was similar to TVC (Figure 1b). The treatment groups had an obviously lower PBC than CK, with SAEW + OG having the lowest at the end of storage. This indicates that SAEW and OG treatments have antimicrobial effects. The results show that the combined treatment was more effective than the single SAEW treatment in reducing microorganisms on salmon fillets. This may be due to the production of Cl_2_ at the anode during the generation of acidic electrolyzed water, which in turn produces HCl and HClO. Among them, HClO has strong antibacterial properties [32]. On the other hand, ozone has strong oxidizing properties that can destroy the cell wall and cell membrane of microorganisms, disrupting the osmotic balance of substances inside and outside the cell, thereby achieving sterilization effects [33].

The TVB-N values (Figure 1c) of all groups showed an upward trend. At the end of storage, the TVB-N values of the SA + OG and OG groups were the lowest (*p* < 0.05). The results showed that SA + OG treatment inhibited the increase in the TVB-N value of salmon fillets to a large extent, and prolonged the shelf life of salmon fillets. Chang, et al. [34] also reached a similar conclusion in their study on the quality change in shrimp after slightly acidic electrolyzed water treatment.

Additionally, the TBARS values (Figure 1d) of the OG group and the SA + OG group were 0.46 and 0.49 mg/kg on day 10, respectively, which were lower than those in the CK group (1.00 mg/kg) (*p* < 0.05). The results showed that OG was superior to SAEW in inhibiting lipid oxidation in salmon fillets. Similar findings were reported by Qian, Zhang, Liu, Ertbjerg, and Yang [7], that it may be due to the antibacterial effect of OG and SAEW.

Figure 1e showed the a* value (redness) in salmon during storage at 4 °C. The redness is a typical appearance of salmon fillet due to astaxanthin, which is an natural antioxidant [35]. The a* value decreased slightly on the first day after treatment (*p* > 0.05), due to the bleaching effect of OG and SAEW to. But at the later stage of storage, the SAEW, OG, and SA + OG had higher a* values than the CK, indicating a lower oxidation degree of astaxanthin. These results indicate that SAEW, OG, and SA + OG treatments effectively inhibited the color change in salmon fillets during storage. Additionally, the SA + OG group had the lowest a* value on day 0, possibly due to the bleaching of salmon fillets caused by the strong oxidation of SAEW. Similar results were found in the study by Mikš-Krajnik et al. [35]. The strong oxidizing properties of OG may also cause astaxanthin to be converted to other colorless compounds.

The TCA soluble peptides in the SA + OG samples were obviously lower (2.125 μmol tyrosine/g muscle) than in the CK, SAEW, and OG samples (4.226, 3.406 and 2.766 μmol tyrosine/g muscle) at the end of storage (Figure 1f), indicating a lower degree of protein degradation. This phenomenon might be related to the antibacterial effect of SAEW and OG, but might also be related to the inhibition effect on endogous enzymes.

Based on the study above, the antibacterial and anti-spoilage effects of ozone and SAEW on the salmon fillets were proved. Therefore, their effects on salmon protein degradation, oxidation, and texture were studied further.

### 3.2. Effect of OG and SAEW on Muscle Properties Analysis

#### 3.2.1. Changes in Texture

**Figure 2:**
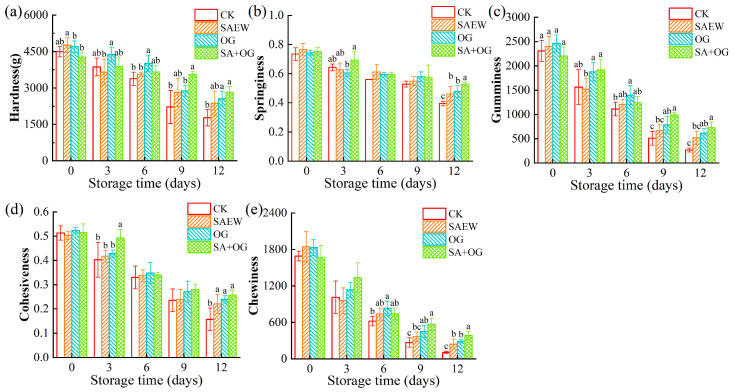
Changes in hardness (**a**), springiness (**b**), gumminess (**c**), cohesiveness (**d**), and chewiness (**e**) of salmon fillets after treatment with ozone gas and slightly acidic electrolyzed water. (Different letters represent significant differences between four groups on the same day (*p* < 0.05)).

Texture changes can directly reflect the structural alterations in the muscle tissue of aquatic products post-mortem, making it an important indicator for reflecting the quality of aquatic products [36]. Hardness is a key indicator of the firmness of the salmon fillet. Springiness reflects the ability to return to its original shape after deformation. Gumminess, cohesiveness, and chewiness are used to measure the energy required to break down the salmon meat, the structural integrity of the salmon fillet, and the complexity of the texture of salmon fillet, respectively [37]. Hardness is considered to be the most important parameter in texture and an important structural characteristic of fish [12]. The hardness values of all groups showed a decreasing trend during storage, and the decrease rate of CK was significantly faster than other groups, as shown in Figure 2a. At the end of storage, the hardness of CK, SAEW, OG, and SA + OG fillets, respectively, decreased by 60.58%, 50.17%, 45.60%, and 33.95%. Springiness had the same trend as the hardness (Figure 2b). At the end of storage, the springiness of CK, SAEW, OG, and SA + OG fillets decreased by 46.19%, 39.93%, 35.75%, and 29.96% in comparison with the originals, respectively. The decrease in hardness and springiness is usually due to the breakdown of proteins by microorganisms and endogenous proteases, leading to the destruction of protein structures [38]. The OG + SA group decreased slowly and was lower than that of the CK, SAEW, and OG groups (*p* < 0.05). This may be because SAEW and OG can inhibit the growth and reproduction of microorganisms, thereby effectively reducing the destruction of the spatial structure of proteins [39].

The gumminess, cohesiveness, and chewiness all showed a downward trend during storage, with CK decreasing the fastest (Figure 2c–e). Both autolysis and the proliferation of microorganisms to muscle degradation may be responsible for the changes in texture [38,39,40].

#### 3.2.2. Changes in Drip Loss

**Figure 3:**
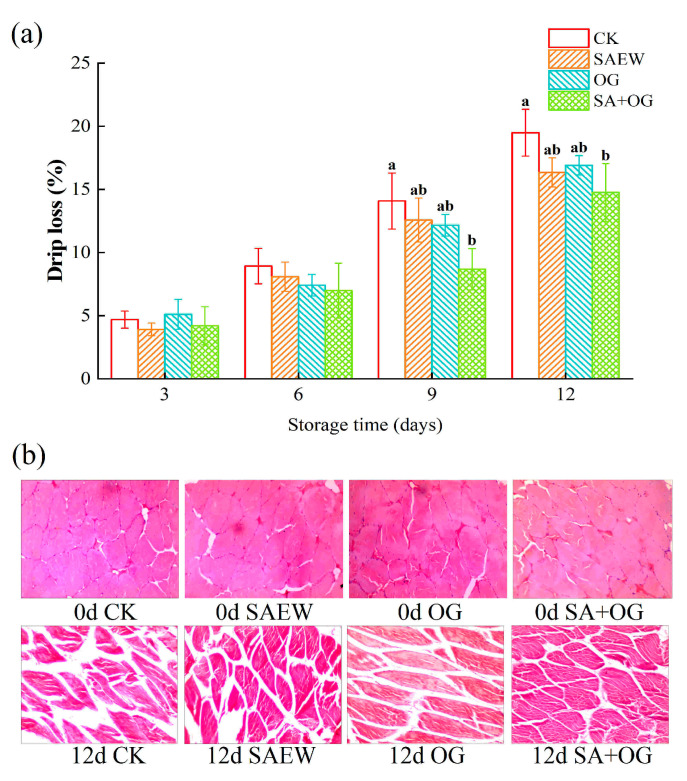
Changes in drip loss (**a**), and microstructural characteristics (**b**) of salmon fillets after treatment with ozone gas and slightly acidic electrolyzed water (Different letters represent significant differences between four groups on the same day (*p* < 0.05)).

Drip loss is related to the fish weight loss and the deterioration of appearance [41]. As shown in Figure 3a, the drip loss of salmon fillets of all groups during storage was increased. The drip loss in the CK group was the highest among all treatment groups, reaching 19.49% on day 12. Qian, Zhang, Liu, Ertbjerg, and Yang [7] have also obtained similar results through a study of the effect of ozone treatment on the quality of salmon. The lowest drip loss was found in the SA + OG group (14.76%), in accordance with the hardness. A less drip loss should be attributed to the inhibition of the degradation of muscle tissue.

#### 3.2.3. Microscopic Observation of the Fillet Tissues

The original microstructure salmon muscle was tightly organized at the beginning of storage (Figure 3b), being regular and full in shape with clear cell borders, which indicated that the salmon samples were fresh [3,42]. The tissue spacing of all groups enlarged during storage. At the end of storage, the CK group displayed the most obvious deformations and irregular ruptures in the muscle tissue and distinct irregular pores in the tissue spacing, indicating the degradation of the protein among the four groups [40]. The muscle tissues of the treated groups were more neatly arranged than the CK group, especially in the SA + OG group. This suggests that SAEW, OG, and their combined treatment (SA + OG group) were effective in inhibiting changes in intertissue proteins. The results were in accordance with the changes of hardness, springiness, and drip loss.

### 3.3. Effects of OG and SAEW on Protein Oxidation

#### 3.3.1. Changes in the Carbonyl Group of Myofibrillar Protein

**Figure 4:**
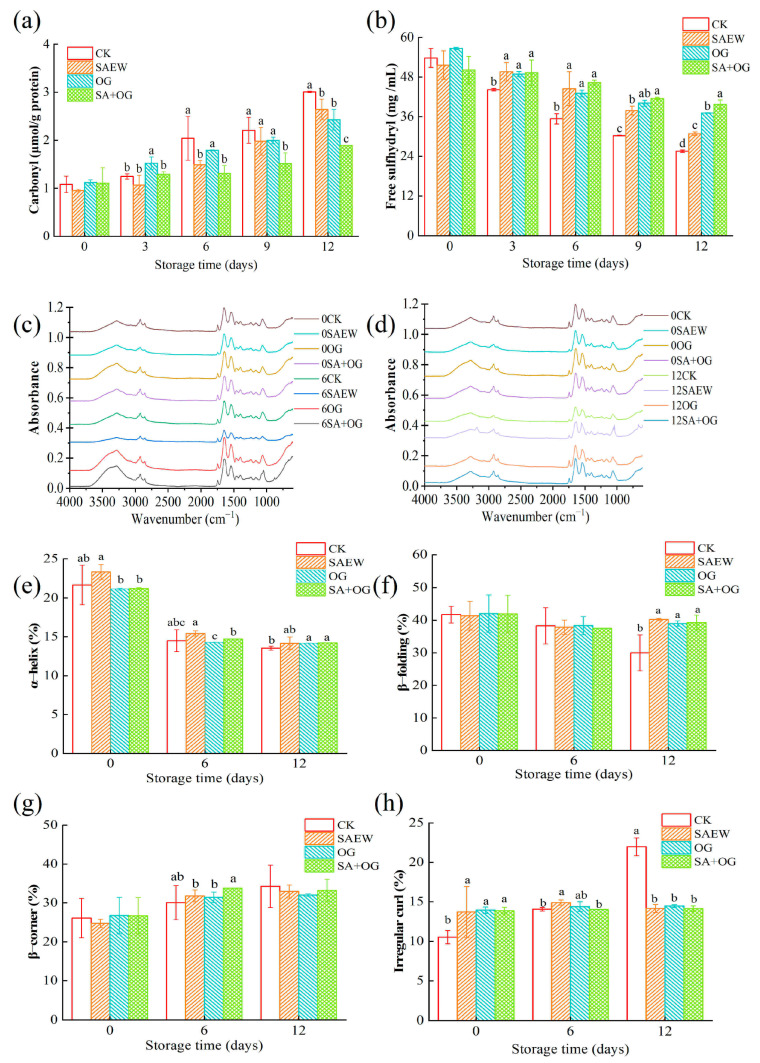
Changes in carbonyl content (**a**), free sulfhydryl content (**b**), and FTIR spectra of salmon fillets (Fresh vs. samples on day 6 (**c**), Fresh vs. samples on day 12 (**d**)); secondary structure content (**e**–**h**) of salmon fillets treated with ozone gas and slightly acidic electrolyzed water (different letters represent significant differences between the four groups on the same day (*p* < 0.05)).

Carbonyl groups are an important indicator for detecting protein oxidation [43]. The carbonyl content (as shown in Figure 4a) of all groups increased significantly with the storage time. Similar results were found in the study of tilapia protein oxidation by Zhao, Zhou, Zhao, Chen, He, and Yang [41]. The carbonyl content of the fresh sample (CK group) on day 0 was 1.08 nmol/mg protein. At the end of storage, the carbonyl content of the CK, SAEW, OG, and SA + OG groups was 3.01, 2.64, 2.43, and 1.90 nmol/mg protein, respectively. This might be due to cross-links formation after the cleavage of the skeleton due to protein oxidation, which converts some amino acid residues into carbonyl derivatives [44]. The results showed that SAEW and OG could effectively inhibit the increased rate in carbonyl content, especially the combined treatment group (SA + OG). This may be due to components such as Cl_2_, HCl, and HClO in SAEW, which have a positive effect on the maintenance of the meat against protein oxidation, in accordance with the previous study [45].

#### 3.3.2. Changes in the Free Sulfhydryl Group of Myofibrillar Protein

During the storage period, there was a reduction in the sulfhydryl content across all groups, as depicted in Figure 4b. This pattern mirrors the findings from Xiong, et al. [46]. The sulfhydryl groups undergo oxidation to form disulfide bridges or other oxidized species, which leads to a reduction in the availability of free sulfhydryl groups [47]. The sulfhydryl content in the control group (CK) dropped from 53.75 nmol/mg protein on the initial day to 25.56 nmol/mg protein on the last day. On day 12, the sulfhydryl content for the SAEW, OG, and SA + OG groups was recorded at 30.85, 37.09, and 39.70 nmol/mg protein, respectively. Thus, the treated groups notably slowed down the decline in sulfhydryl content, with the SA + OG group showing the most effectiveness. It is noteworthy that the OG group exhibited a higher sulfhydryl content than the CK group on day 0, potentially due to ozone altering the structure of the protein, making previously concealed sulfhydryl groups accessible on the surface of proteins. Similar observations were made by Zhang, et al. [48] in their study on the impact of ozone on the physicochemical properties of bighead carp meat.

#### 3.3.3. FT-IR Analysis of Protein Structures

The wavenumber and absorbance of the FTIR spectrum can be used to represent the secondary structure of proteins. The main band in myofibril (amide I, 1600–1700 cm^−1^) is considered to be the most important band for analyzing the secondary structure of proteins [49] because it is sensitive to hydrogen bond distribution, dipole–dipole interaction force, and changes in peptide skeleton structure [41,50,51]. In general, the spectral peaks of all samples from day 0 to day 6 did not change significantly (Figure 4c). However, the spectral peaks of each group on day 12 (Figure 4d) lowered, which may be due to the destruction of hydrogen bonds between proteins [52]. On day 12, the spectral peaks of SA + OG and OG were most similar to the original sample, indicating that the secondary structure changes the least. This might be due to the fact that the combination of OG and SAEW significantly reduced the protein oxidation degree and the damage to the secondary structure of proteins.

As shown in Figure 4e–h, α-helix showed a significant decrease in the middle stage of storage. The percentage of β-folding in the CK group was significantly higher than SAEW, OG, and SA + OG groups, while the percentage of an irregular curl in the CK group was significantly higher at the end of storage (*p* < 0.05). This may be due to the oxidation and denaturation of the protein breaking the hydrogen bonds in the protein, exposing more hydrophobic groups. These hydrogen bonds are essential for maintaining the stability of α-helix and β-fold structures. The disruption of hydrogen bonds leads to the disintegration of the structure, and the interaction of hydrophobic groups leads to structural instability and the rearrangement of proteins, thereby increasing the content of irregular curl and β-turns [41]. In addition, the acidic environment created by SAEW may also alter the protein structure, resulting in an increase in the content of β-fold [51]. The treated groups showed a significantly higher proportion of β-folding and a lower proportion of irregular curl (*p* < 0.05) than the control. Therefore, the treatments could maintain the structure of proteins and prevent the rearrangement of proteins.

### 3.4. Effects of OG and SAEW on Protein Degradation

#### 3.4.1. SDS-PAGE Profile

**Figure 5:**
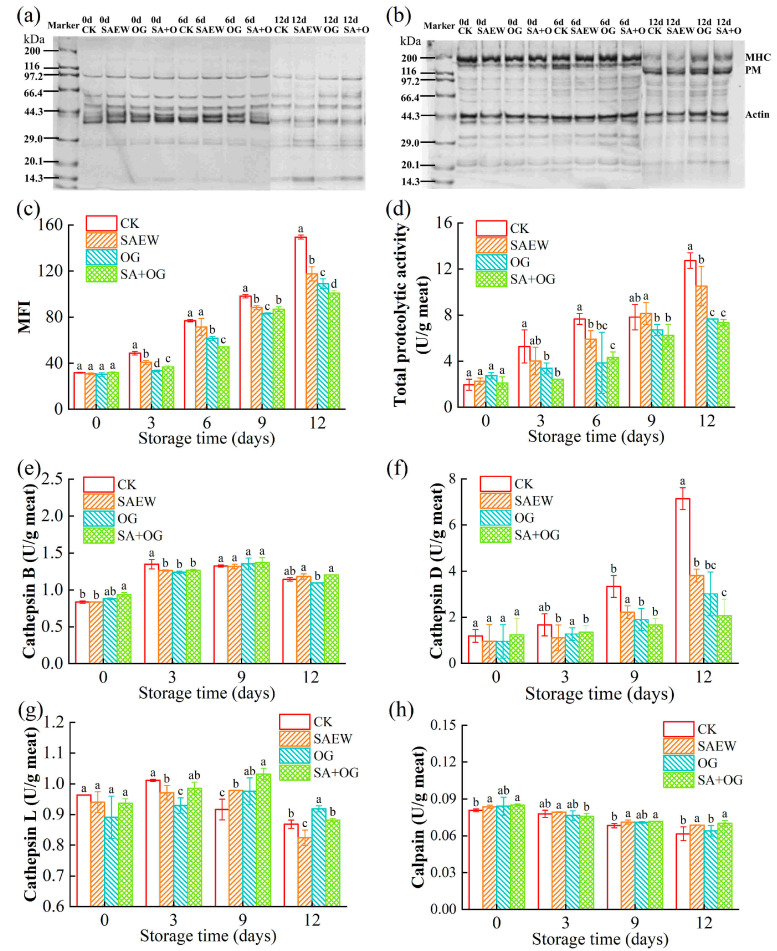
SDS-PAGE profiles of sarcoplasmic proteins (**a**) and myofibrillar proteins (**b**), the changes of MFI values (**c**), total proteolytic activity (**d**), cathepsin B activity (**e**), cathepsin D activity (**f**), cathepsin L activity (**g**), and calpain activity (**h**) of salmon fillets after treatment with ozone gas and slightly acidic electrolyzed water (different letters represent significant differences between the four groups on the same day (*p* < 0.05)).

In this experiment, we mainly studied a water-soluble sarcoplasmic protein and salt-soluble myofibrillar protein. Sarcoplasmic proteins are mainly metabolism-related enzyme proteins, and their molecular weights are mainly distributed in the range of 20~200 kDa [53]. Salt-soluble proteins are mainly myofibrillar proteins, with molecular weights mainly ranging from 14 to 200 kDa, including myosin heavy chain (MHC, ~200 kDa), paramyosin (PM, ~100 kDa), actin (AC, ~44 kDa), tropomyosin (TM, ~35 kDa), and myosin light chain (MLC, <20 kDa) [27,54]. As shown in Figure 5a, there was no obvious change in sarcoplasmic protein bands in all groups on day 0 and 6, but the intensity of the bands was decreased obviously on day 12, especially in the CK and SAEW groups. At the end of storage, the intensity of the band around 29.0 kDa increased in all groups, and a new band appeared at 14.3 kDa. This phenomenon indicated the degradation of the sarcoplasmic protein, probably due to bacterial activity. The bands of the myofibrillar protein electropherograms on days 0 and 6 also did not show obvious changes (Figure 5b). At the end of storage, all groups showed a significant weakening of the MHC bands, especially in the CK group where the MHC bands almost disappeared. New bands appeared at 116–200 kDa, which may be due to the degradation of myosin [55]. It was demonstrated that OG and SAEW + OG significantly inhibited the degradation of myogenic fibronectin.

#### 3.4.2. Changes in MFI

MFI is an important indicator of myofibrillar integrity [54]. As shown in Figure 5c, the MFI values of each group increased with the extension of storage time. At the beginning of storage, there was no significant difference among the groups, but at the end of storage, the MFI value of the CK group was significantly higher than that of the SEAW, OG, and SA + OG groups, in agreement with the tendency of TCA-soluble peptides. The activity of endogenous enzymes and the microorganisms are the main reasons for the degradation of myofibrillar protein and the increase in the MFI value [13]. The OG and SAEW could significantly inhibit the increase in the MFI value. This may be contributed to the inhibitory effect of SAEW and OG against endogenous proteolytic activity and microbial growth. Similar results have been found in the study of the effect of slightly acidic electrolyzed water on the quality of aquatic products [13,56].

#### 3.4.3. Changes in Total Proteolytic Activity

The total proteolytic activity showed an overall upward trend with the extension of storage time (Figure 5d). There was no significant difference in the total proteolytic activities on day 0, and the increase in the CK group was significantly higher than that in the SAEW, OG, and SA + OG groups on the third day. At the end of storage, the total protease activity of the treatment groups, was significantly lower than the CK group. This may be due to the fact that proteases are sensitive to oxidants. HClO in SAEW can destroy the structure of some proteases through oxidation [51]. Similarly, the strong oxidizing nature of OG can also change the natural structure of the protease, which reduces the activity of the protease [41]. In addition, SAEW and OG strongly inhibited the growth of microorganisms, whose proliferation also accelerates the secretion of proteases [57], thereby inhibiting the activity of proteases.

#### 3.4.4. Changes in Cathepsin Activity

Cathepsin B showed an increasing trend in the first stage and a decreasing trend afterwards, which reached a peak on day 3 (Figure 5e). Yu, Regenstein, Zang, Xia, Xu, Jiang, and Yang [26] showed a similar trend in the effect of a chitosan coating at 4 °C on the endogenous proteolytic activity of stored grass carp fillets. The activities of cathepsin B increased after OG and SAEW treatments but became lower than the CK group on day 3. Therefore, the activity of cathepsin B was inhibited by the treatments. This may be due to the strong oxidizing properties of OG and SAEW, which inhibit the activity of cathepsin B by destroying the natural structure [58]. It is reported that cathepsin B plays an important role in the spatial structure change in protein and the texture change in fish tissues [59].

Cathepsin D is active in an acidic environment (pH 3.0~5.0), and has a greater effect on the heavy chain of myosin [60]. With the extension of storage time, the activity of cathepsin D continued to increase (Figure 5f). The activity of cathepsin D of the four groups on day 0 was close to each other. The higher the activity of cathepsin D, the more severe the degradation of myofibrillar protein and the deterioration of fish tissue texture. The SAEW group showed a lower activity of cathepsin D than the CK group, but higher than OG and SA + OG. This may be due to the fact that cathepsin D is not sensitive to the acidic environment created by SAEW compared with other enzymes [58], and OG displayed a higher inhibitory effect. SA + OG showed the lowest cathepsin D activity during storage, indicating that the combined treatment had a synthetic effect.

As shown in Figure 5g, the activity of cathepsin L in CK increased from day 0 to day 3 but decreased afterwards. Additionally, the activities of cathepsin L decreased after the treatments on day 0, and gradually increased until day 9. It seems that the treatments of OG and SAEW delayed the changes of its activity rather than inhibited it. According to the activity data of the three enzymes, it seems that cathepsin D was more sensitive to SAEW and OG than the others.

#### 3.4.5. Changes in Calpain Activity

Calpain is a proteolytic enzyme that affects the structure of myofibrillar proteins, resulting in a softening of the texture of fish tissues. The activity of calpain decreased slightly during storage (Figure 5h). However, there was no significant difference in calpain activity between the CK group and the treatment group during storage. It seemed that calpain was not sensitive to OG and SAEW.

### 3.5. Results of Correlation Analysis

**Figure 6:**
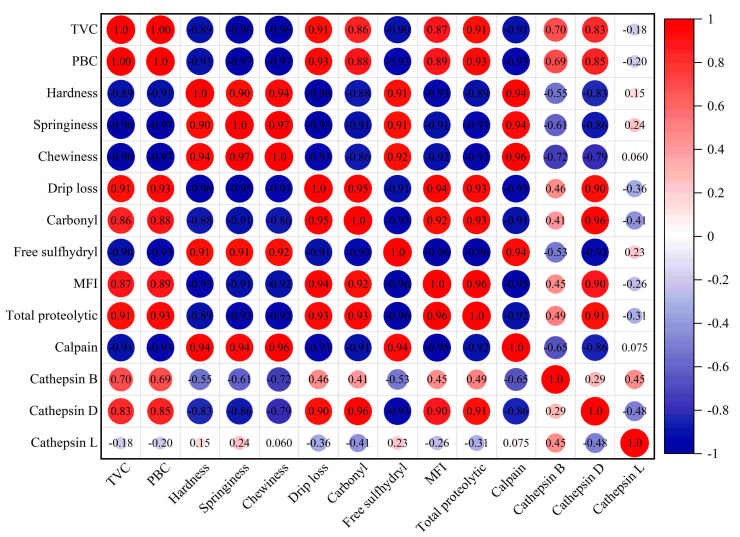
Correlation analysis of texture characteristics, protein oxidation and degradation, and endogenous proteases in salmon treated with ozone gas and slightly acidic electrolyzed water.

Figure 6 shows that the MFI and carbonyl content were strongly negatively correlated with hardness, springiness, chewiness, and drip loss, while the free sulfhydryl content was strongly positively correlated with these indicators. It demonstrated that the changes of texture properties and drip loss should be contributed to protein degradation and oxidation [61]. Besides, the TVC, PBC, the total proteolytic activity, and the activities of cathepsin D and B were negatively correlated with hardness, springiness, chewiness, and the free sulfhydryl content, and positively correlated with the MFI, carbonyl content, and drip loss. The activity of cathepsin L was less correlated with the texture, drip loss, and MFI, while calpain had a negative relationship with drip loss, and a positive correlation with texture properties including hardness, springiness, and chewiness. It demonstrated that both bacteria and cathepsin D and B should be the main factors responsible for protein degradation and oxidation, consequently leading to texture changes in salmon.

### 3.6. Mechanisms of OG and SAEW on Muscle Changes in Salmon

**Figure 7:**
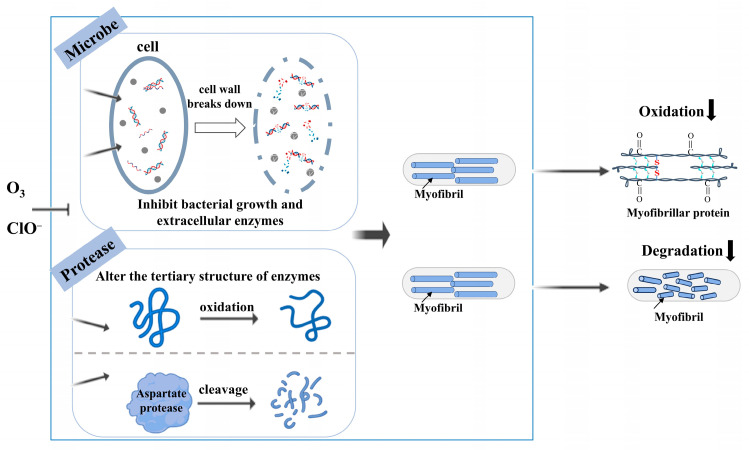
Mechanism diagram of ozone gas and slightly acidic electrolyzed water.

According to the study above, the possible mechanism of OG and SAEW is concluded in Figure 7. The bacteria and cathepsin D and B should be the main factors for the texture quality deterioration of salmon. On one hand, OG and SAEW inhibited the growth of bacteria, leading to a lower secretion of extracellular protease and lower degree of protein oxidation. On the other hand, OG and SAEW reduced the activity of endogenous proteases, especially cathepsin D, which is responsible for the degradation of myofibrillar protein.

## 4. Conclusions

In summary, this study demonstrated that the combined treatment of ozone gas (OG) and slightly acidic electrolyzed water (SAEW) effectively retarded the degradation of muscle proteins in salmon during chilled storage. Microscopic observations provided visual evidence of improved tissue integrity in the SA + OG treated group. Additionally, the combined treatment inhibited the protease activities, especially cathepsin D. The results indicated that the combination of OG and SAEW also provided better protection against protein oxidation compared to the control or single-treatment groups. Correlation analysis further confirmed the role of these enzymes in the quality loss of salmon fillets. Overall, this study provided an insight into the application of OG and SAEW for maintaining the texture and freshness of salmon by targeting the endogenous proteases and bacteria. However, more studies related to how OG and SAEW affect lipid oxidation should be confirmed, as well as the structure of the protein by X-ray assay.

## Data Availability

The original contributions presented in the study are included in the article, further inquiries can be directed to the corresponding author.
